# Light enhanced the antimicrobial, anticancer, and catalytic activities of selenium nanoparticles fabricated by endophytic fungal strain, *Penicillium crustosum* EP-1

**DOI:** 10.1038/s41598-022-15903-2

**Published:** 2022-07-12

**Authors:** Amr Fouda, Saad El-Din Hassan, Ahmed M. Eid, Mohamed Ali Abdel‐Rahman, Mohammed F. Hamza

**Affiliations:** 1 Department of Botany and Microbiology, Faculty of Science, Al‐Azhar University, Nasr City, 11884 Cairo Egypt; 2 Nuclear Materials Authority, P.O.B. 530, El-Maadi, 11728 Cairo Egypt; 3 School of Nuclear Science and Technology, University of South China, Hengyang, 421001 China

**Keywords:** Biological techniques, Biotechnology, Cancer, Microbiology, Environmental sciences, Nanoscience and technology

## Abstract

Selenium nanoparticles (Se-NPs) has recently received great attention over owing to their superior optical properties and wide biological and biomedical applications. Herein, crystallographic and dispersed spherical Se-NPs were green synthesized using endophytic fungal strain, *Penicillium crustosum* EP-1*.* The antimicrobial, anticancer, and catalytic activities of biosynthesized Se-NPs were investigated under dark and light (using Halogen tungsten lamp, 100 Watt, λ > 420 nm, and light intensity of 2.87 W m^−2^) conditions. The effect of Se-NPs was dose dependent and higher activities against Gram-positive and Gram-negative bacteria as well different *Candida* spp*.* were attained in the presence of light than obtained under dark conditions. Moreover, the viabilities of two cancer cells (T47D and HepG2) were highly decreased from 95.8 ± 2.9% and 93.4 ± 3.2% in dark than those of 84.8 ± 2.9% and 46.4 ± 3.3% under light-irradiation conditions, respectively. Significant decreases in IC_50_ values of Se-NPs against T47D and HepG2 were obtained at 109.1 ± 3.8 and 70.4 ± 2.5 µg mL^−1^, respectively in dark conditions than 19.7 ± 7.2 and 4.8 ± 4.2 µg mL^−1^, respectively after exposure to light-irradiation. The photoluminescence activity of Se-NPs revealed methylene blue degradation efficiency of 89.1 ± 2.1% after 210 min under UV-irradiation compared to 59.7 ± 0.2% and 68.1 ± 1.03% in dark and light conditions, respectively. Moreover, superior stability and efficient MB degradation efficiency were successfully achieved for at least five cycles.

## Introduction

Nanomaterials are characterized by their particles in the nano-size range (1–100 nm), with a high surface-to-volume ratio. Moreover, the quantum, surface, tinny volume, and interface effects differentiate them than their bulk metal counterparts with innovative chemical and physical properties^1,2^. Nanoparticles (NPs) exhibit high properties in strength, mobility, reactivity, and solubility, and therefore NPs are suitable for several engineering, biological, medicinal, and environmental applications^3–6^. In addition, the green synthesis (bio-fabrication) of nanomaterials with microorganisms, plants, or their extracts is attractive for avoiding chemical risk, high temperatures/pressures, and the addition of external capping and stabilizing agents^3^. Besides that, it is a cost-effective, biologically safe, and environmentally friendly option^7^. Amongst microorganisms, fungi are the optimal and most beneficial choice for NPs bio-fabrication due to their easy handling in the laboratory, providing high binding strength, and great ability to accumulate and tolerate metals intracellularly. In addition, the fungal mycelia can combat agitation and pressure flow in bioreactors and secrete large amounts of enzymes for reducing metal ions to form metallic NPs^1^.

Selenium (Se) is one of the essential elements for humans, animals, and plants. It has an effective role in plant growth and protects it from various stresses^8^. As it is a semi-conductor and photoelectrically active, it has more advanced applications. Selenium is renowned in semiconductor devices, solar cells, and photographic films because of their unique properties such as optical and thermal conductivity, and sensing for X-rays^9,10^. Metal NPs of Au and Ag, have immense several applications but are costlier to synthesize than Se nanoparticles (Se-NPs). Additionally, Se-NPs have tremendous applications in the biomedical field and can be integrated with other biological agents to enhance their biological properties. The Se-NPs offer higher biocompatibility and lower toxicity than either inorganic or organic Se compositions. Therefore, great attention has been oriented for Se-NPs implementation as therapeutic agents, anticancer, biocidal, antioxidant, catalytic, and photoreactive properties. In addition to their potential actions as antimicrobial coatings, nutritional supplements, medical devices, diagnostics; other applications such as solar cells, photocopiers, rectifiers, and xerography have been reviewed^10–12^. In addition, Se-NPs have multilateral physical properties; for instance, their ability to form various shapes depending on the fabrication approaches and stabilization agents^11^.

The biogenic Se-NPs manifested potential broad-spectrum activity against Gram-positive and Gram-negative bacteria, unicellular, and multicellular fungi^13^. This behavior may be attributed to the overproduction of reactive oxygen species (ROS) that causing cell membrane damage, inhibiting the amino acids synthesis, and blocking DNA replication^14,15^. The potency of Se-NPs to regulate free radicals and induce autophagy or apoptosis has been exploited as an anti-cancer therapy in addition to ridding host cells of pathogens. Moreover, the action of these nanoparticles as delivery carriers to encapsulate biomolecules or drugs increases their efficacy as a direct killer of cancer cells^16^. Besides these activities, Se-NPs have been explored for the treatment of various diseases; for instance, diabetes, rheumatoid arthritis, and Alzheimer’s disease. Also, Se-NPs can be used as a protective agent against different toxic substances such as cadmium, chromium, and other chemotherapeutic agents that have negative impacts on health^17,18^. Interestingly, the activity of Se-NPs can be increased due to modification of its surface. For instance, the functionalized modification of the surface of Se-NPs fabricated by aqueous extract of *Artemisia annua* with starch to form St-SeNPs exhibit high antibacterial activity against *Escherichia coli, Salmonella enterica, Staphylococcus aureus,* and *Bacillus cereus* compared to original Se-NPs^19^.

Azo and thiazine are chemically stable dyes which have been discharged directly to the ecosystem through industrial effluent. These dyes are highly toxic and considered ones of the main sources of environmental pollution. These dyes are mutagenic, carcinogenic, with teratogenic nature for humans, animals, plants, and aquatic biota^20,21^. Therefore, it is urgent to develop an innovative method characterized by simplicity, efficiency, low cost, and rapid oxidation with low toxic by-product productions. The photocatalytic technique should facilitate these advantages to overcome the disadvantages of traditional methods^22^.

Photocatalysis means the accelerated photoreaction in presence of catalysts. Recently, NPs have been used as catalysts due to their remarkable large surface area, high aspect ratios, exceptionally reliable, electrostatic and magnetic properties, compressible without modifying their surface area, hydrophilic and hydrophobic characteristics, and tunable pore volume^23,24^. The initial step of this technique begins with light (sunlight, halogen light, or UV) has been absorbed by a catalyst that enhances the production of electron–hole pairs which interact with the substrate to generate free radicals. The activity and rate of the reaction are dependent on the efficacy of the catalyst in producing these electron–hole pairs. After that, free radicals react with the pollutants to form various useful less toxic products. In the case of living entities (i.e. bacteria, viruses, fungi, etc.), the formed free radicals enhance the overproduction of ROS which lead to cell death^22,25^.

Endophytic microbes including bacteria, fungi, and actinomycetes are characterized by high secretion of active metabolites that can be used to fabricate NPs with varied sizes, shapes, and high stability^26^. Endophytic fungi are considered more active than non-endophytic species, either in the number of producing metabolites or the activity of these metabolites^27^. Therefore, the activity of these fungal strains and their metabolites should be explored for possible use in biomedical and biotechnological applications. Herein, Se-NPs were fabricated using endophytic fungal strain, *Penicillium crustosum* as a green approach. The Se-NPs characters were investigated using color change, UV–Vis spectroscopy, X-ray diffraction (XRD), Fourier transform infrared (FT-IR), Transmission Electron Microscopy (TEM), and Scanning Electron Microscopy connected with energy-dispersive X-ray (SEM–EDX). The comparative study between the activity of green synthesized Se-NPs under dark and light irradiation conditions were investigated. The antimicrobial and anticancer activities against human breast cancer cell lines (T47D) and human liver cancer cell lines (HepG2), as well the catalytic activity against methylene blue dye were investigated in the presence and absence of light irradiations. Few studies were concerned by comparative investigation between the various activities of NPs at dark and light conditions. To the best of our knowledge, this is the first report on the green synthesis of Se-NPs using *P. crustosum*; hence, the environmental and applicable importance of this study has gained from the safety nature of fungal endophyte strain.

## Results and discussion

### Green synthesis of Se-NPs

In the current study, Se-NPs were fabricated by harnessing the active metabolites secreted by the endophytic fungal strain, *Penicillium crustosum* EP-1 for reducing and capping the Na_2_SeO_3_ to form Se-NPs. At the first, the successful fabrication was confirmed by the color change of fungal biomass filtrate after mixing with metal precursor from colorless to ruby red color. The formed color was gradually increased with time as a result of SeO_3_ reduction; therefore, we checked the formed color and measured its absorbance after 24 h of incubation. After that, no further color change was found and this depicts the complete reduction of SeO_3_ to Se^0^. Compatible with this finding, the absorbance of NPs with times was increased but did not shift the SPR peak, this is due to the complete reduction of metal ions to metal NPs and hence increasing the color intensity as reported previously^28–30^. The color of Se-NPs synthesized by *Trichoderma atroviride* was completely formed after incubation of fungal biomass filtrate with a metal precursor for 24 h^31^. In a recent study, the extracellular metabolites secreted by *P. chrysogenum* strain F9 isolated from historical manuscript were used as a biocatalyst to reduce selenium ions forming Se-NPs^8^. The synthesis conditions especially pH values exhibited a critical role in the green synthesis process. Herein, the synthesis of Se-NPs was better in an alkaline medium (pH = 8) because various functional groups in fungal biomass filtrate that are responsible for reduction and capping were more active in alkaline conditions. Also, the alkaline medium prevents the agglomeration of NPs as well as stabilize the capping agents on the NPs surfaces via reacting with amino groups on the protein surfaces^32,33^.

The green synthesis of NPs using fungi has gained more attention than other microorganisms. This is mainly due to the exceptional secretion of extracellular metabolites that increase the NPs yield and impart high stability to the synthesized NPs^34^. Also, fungi are characterized by easy handling, high metal tolerance, high biomass production, and high scalability^35^. The genus *Penicillium* is composed of approximately 354 species that are characterized by producing a wide range of metabolites^36^. In this study, the endophytic strain *P. crustosum* EP-1 was selected for Se-NPs production because of its high efficacy in producing a wide variety of metabolites including enzymes (amylase, cellulase, gelatinase, and xylanase), bioactive metabolites as previously reported^37^. Therefore, we predicted the potentiality of this strain to form small size and highly stable Se-NPs.

### Characterizations of Se-NPs

**Figure 1:**
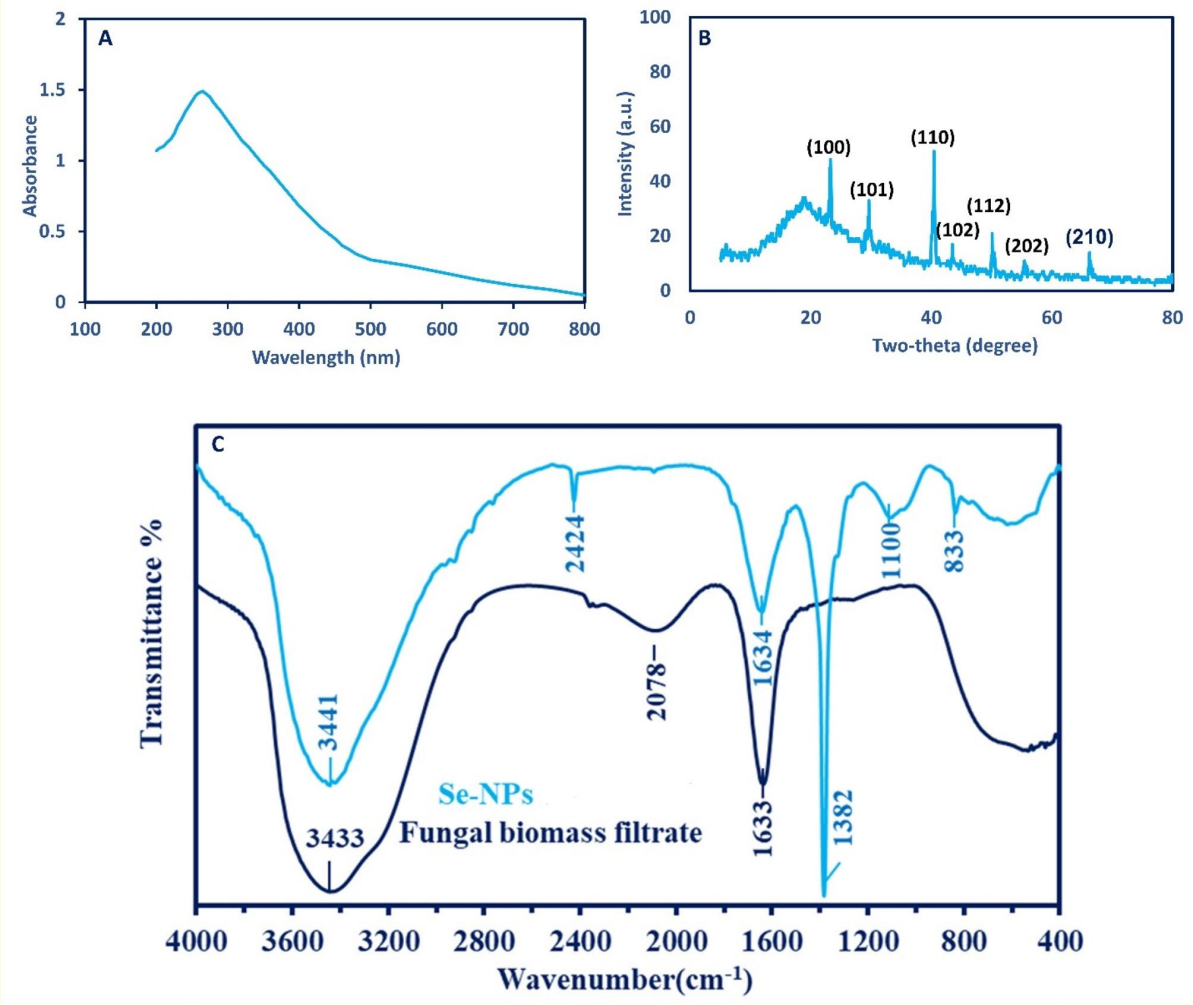
(**A**) UV–Vis spectroscopy that showed the maximum SPR at 270 nm; (**B**) XRD analysis shows the crystallinity nature of green synthesized Se-NPs; and (**C**) FT-IR chart that refer to the various functional groups in fungal biomass filtrate and synthesized Se-NPs.

The first monitor for the successful formation of Se-NPs is the ruby red color that is substantiated by measuring its absorbance using UV–Vis spectra. Data showed that the high absorption peak was at 270 nm (Fig. 1A) and this peak formed and characterized the SPR of Se-NPs. The wide SPR peak mainly reveals the polydispersity of green synthesized Se-NPs. Compatible with our study, the SPR of Se-NPs synthesized by bacterial strain *Ralstonia eutropha* was at 270 nm^30^. The maximum SPR peak of Se-NPs synthesized by aqueous extract of *Ceropegia bulbosa* was observed at 277.5 nm^25^. Recently, the water extract of *Cirsium setidens* was changed from yellow green to brick red color after mixing with Na_2_O_3_Se indicates the formation of Se-NPs that exhibit maximum absorption peak at 278 nm which is correlated to SPR of Se-NPs^38^. Various researchers have reported that the SPR peak in the ranges of 200–300 nm is related to the surface plasmon vibration of Se-NPs^31,39^.

The crystalline nature of formed Se-NPs was confirmed by an X-ray diffractometer as shown in Fig. 1B. Data showed that the Se-NPs X-ray plans of (100), (101), (110), (102), (112), (202), and (210) were matched the Bragg reflection of two theta values of 23.17°, 29.63°, 40.24°, 43.48°, 50.09, 55.51°, and 66.19°, respectively. These Bragg’s diffraction peaks were matched with the JCPDS standard card (No. 06-0362) that confirms the crystalline form of Se-NPs^25,39^. The presence of diffraction peaks at two thetas of 23.17°, 29.63°, and 43.48° of Se-NPs fabricated by endophytic fungal strain indicates the presence of selenium as a result of reduction of SeO_3_ as previously reported^40^.

The roles of different functional groups that exist in the fungal biomass filtrate in the reduction and stabilization of Se-NPs were demonstrated through a comparison of the FT-IR spectra (Fig. 1C). The peak at 3433 cm^−1^ was assigned to O–H and N–H overlapping vibration^41^, this peak was shifted to 3441 cm^−1^ with the same intensity as Se^0^ was fabricated. The broadband at 2078 cm^−1^ of the biomass filtrate was assigned mainly to the sulfonic acid groups (from the medium) and in the carbohydrate moiety (verifying by EDX analysis). The peaks at 1633 cm^−1^ and 1634 cm^−1^ were assigned to the C=O overlapped with the N–H stretching vibration of the produced polysaccharides. Extra peaks that appeared as Se manufactured including 2424 cm^−1^ for S–H stretching bands, and 1382 cm^−1^ for deformation of NO_3_ stretching, 1100 cm^−1^ for C–O–C asymmetric stretching and C–N stretching, while 833 cm^−1^ for C–O–S stretching overlapped with nitrate ions^42^.

**Figure 2:**
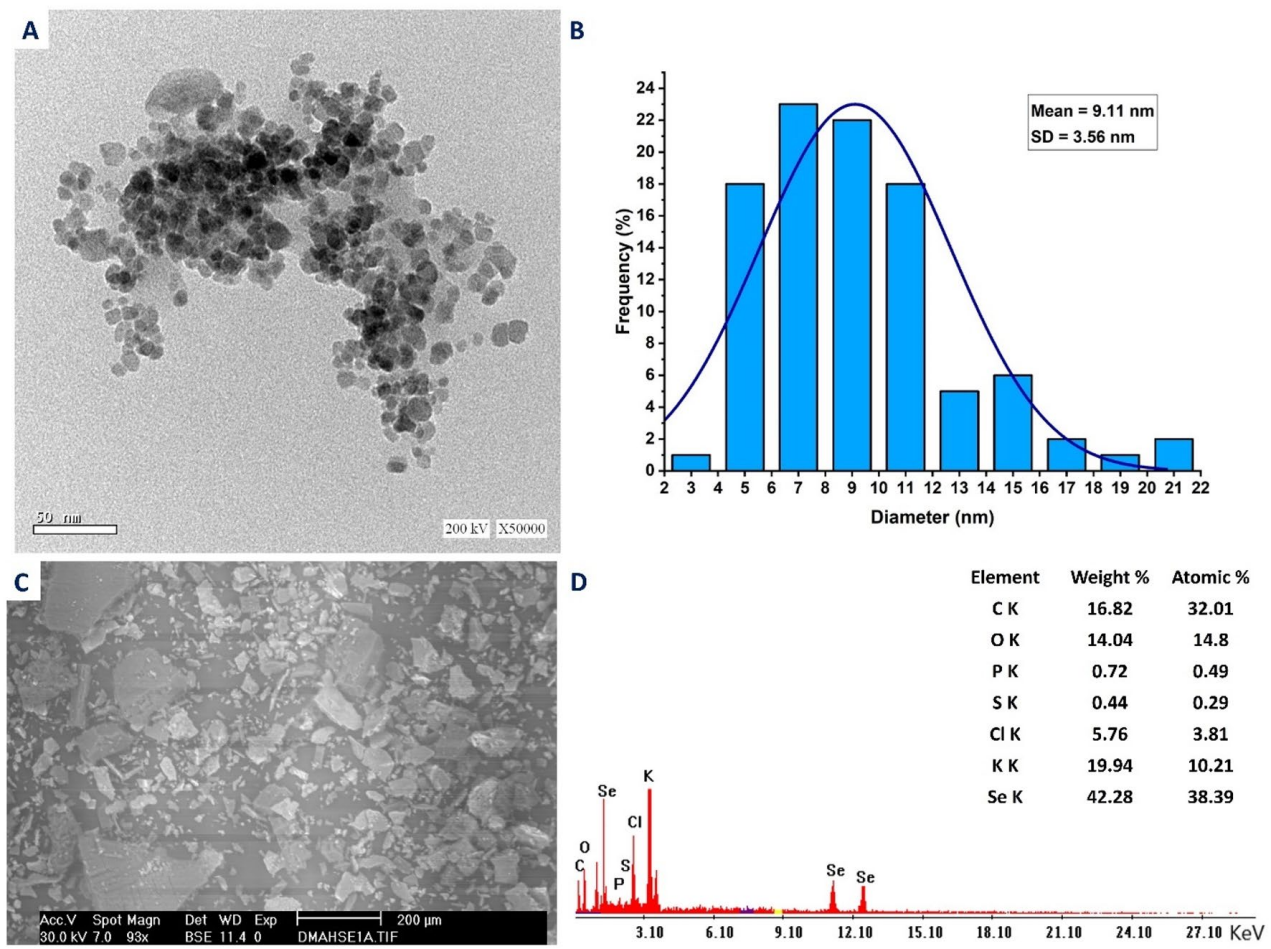
(**A**) TEM image of synthesized Se-NPs showed spherical shape; (**B**) size distribution of NPs based on TEM image; (**C**) and (**D**) SEM–EDX showed the composition of the sample.

There are several parameters determining the incorporation and activities of NPs in specific biomedical and biotechnological applications. These include shapes, coating agents, sizes, elemental compositions, solubility, zeta potential, and aggregation values^43,44^. Therefore, TEM, SEM, and EDX analyses were investigated for the bio-fabricated Se-NPs. As shown in Fig. 2A,B, the green synthesized Se-NPs were a spherical shape, well dispersed, without aggregation and agglomeration, and have a sizes range of 3–22 nm with an average particle size of 9.11 ± 3.56 nm.

The activity of Se-NPs is mainly dependent on their size and always exhibits increment with smaller sizes. For example, the antioxidant activity of Se-NPs fabricated by *Pantoea agglomerans* showed a promising result for a smaller size than the large one^45^. Moreover, Se-NPs synthesized by aqueous extract of garlic showed high antimicrobial activity with sizes of 21– 40 nm than the activity showed by sizes of 41–50 nm^46^. Due to obtained small Se-NPs sizes in the current study, it can be integrated into various activities.

The morphology and chemical compositions of green synthesized Se-NPs, were analyzed by SEM–EDX (Fig. 2C,D). As shown the absorption peaks of Se were identified at 1.38 keV, 11.24 keV, and 12.41 keV for SeLα, SeKα, and SeKβ, respectively. The obtained data are compatible with those reported that the characteristic absorption peaks of Se-NPs synthesized by aqueous extract of *Vitis vinifera* were at 1.37 keV, 11.22 keV, and 12.49 keV for SeLα, SeKα, and SeKβ, respectively^47^. Also, the absorption peaks of Se-NPs fabricated by the bacterial strain *Bacillus cereus* were observed at 1.37 keV, 11.22 keV, and 12.49 keV for SeLα, SeKα, and SeKβ, respectively^9^. The EDX chart showed that the Se was represented by the highest weight element in the sample with percentages of 42.28 and atomic percentages of 38.39 (Fig. 2D). Other weak peaks such as S and Cl were represented in the EDX chart with weight percentages of 0.44 and 5.76%, respectively. These peaks can be related to glass underlay as reported previously^10^. In addition, peaks of C, O, P, and K were present with weight and atomic percentages of (16.82, 14.04, 0.72, and 19.94%) and (32.01, 14.8, 0.49, and 10.21%), respectively. These signals can be originated from fungal biomolecules such as enzymes and proteins that cap and impart the stability of Se-NPs^48^.

### Antimicrobial activity of Se-NPs

**Figure 3:**
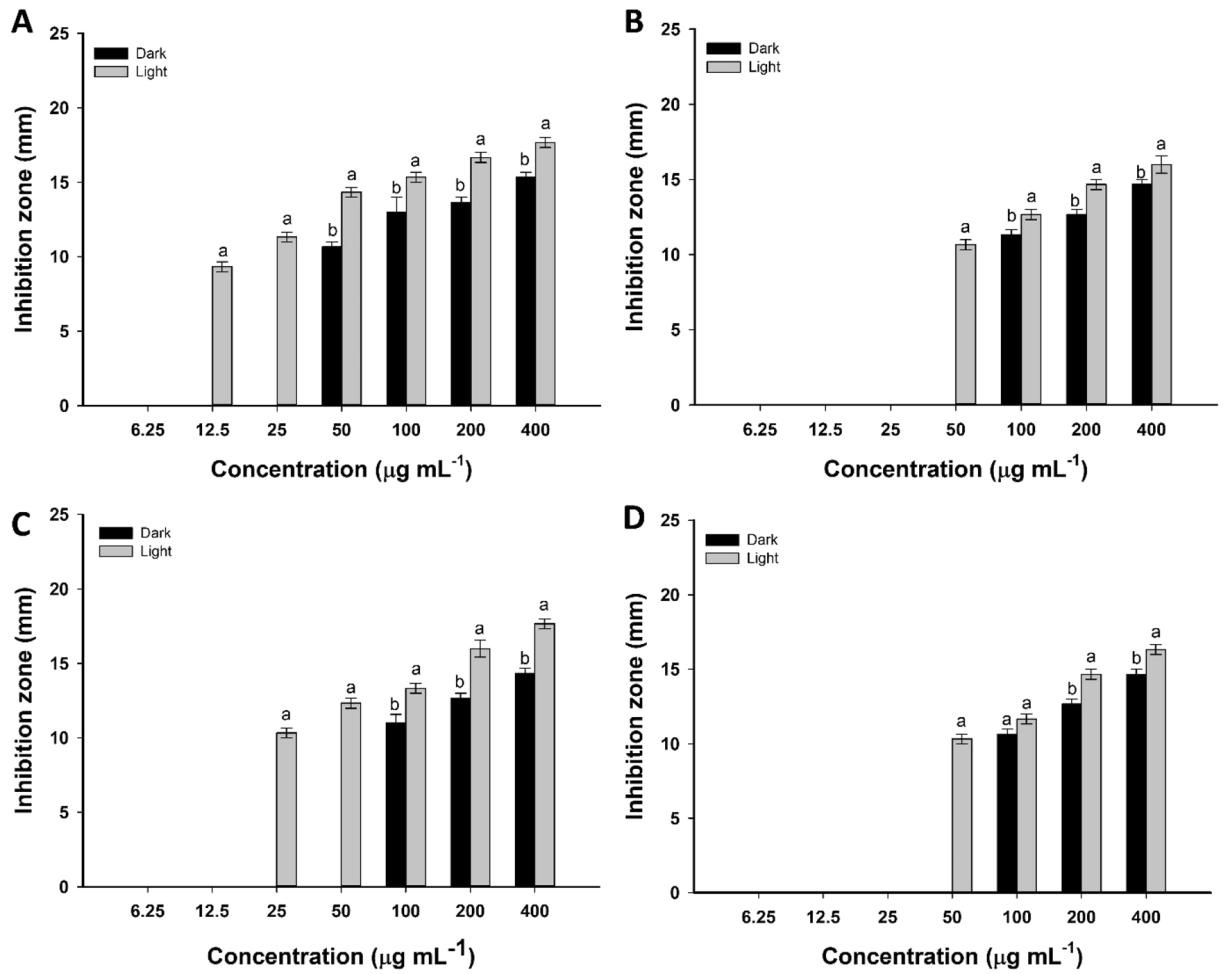
Antibacterial activity of green synthesized Se-NPs against different Gram-positive and Gram-negative bacteria after incubation under dark and light conditions. *Bacillus subtilis* (**A**); *Staphylococcus aureus* (**B**); *Pseudomonas aeruginosa* (**C**)*;* and *Escherichia coli* (**D**)*.* Data are statistically different at *p* ≤ 0.05 by Tukey’s test, (*n* = 3); error bars are based on three independent experiments (means ± SD). Different letters at the same concentration refer to the data that are significantly different.

**Figure 4:**
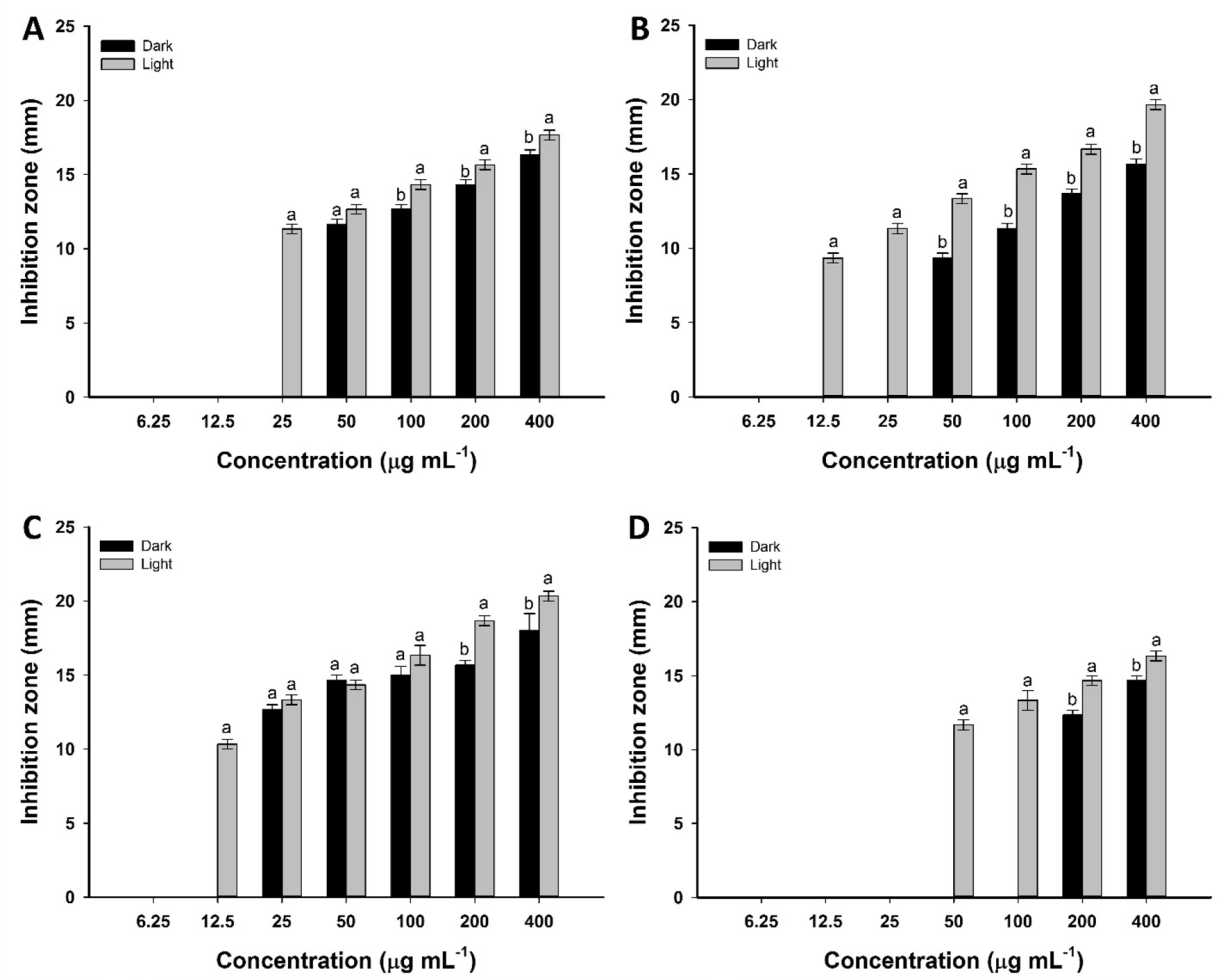
The activity of green synthesized Se-NPs against different strains of *Candida* after incubation under dark and light conditions. *Candida albicans* (**A**); *Candida glabrata* (**B**)*; Candida tropicalis* (**C**)*;* and *Candida parapsilosis* (**D**)*.* Data are statistically different at *p* ≤ 0.05 by Tukey’s test, (*n* = 3); error bars are based on three independent experiments (means ± SD). Different letters at the same concentration refer to the data that are significantly different.

Figures 3 and 4 displayed the antimicrobial efficacy of green synthesized Se-NPs under dark and light conditions against Gram-positive bacteria, Gram-negative bacteria, and unicellular fungal strains at different concentrations. The results exhibited that the activity of synthesized Se-NPs was concentration dependent. Data analysis showed that the diameter of clear zones decreased as Se-NPs concentrations decreased. At high Se-NPs concentration (400 µg mL^−1^), the inhibition zones formed after incubation of *Bacillus subtilis*, *Staphylococcus aureus*, *Pseudomonas aeruginosa,* and *Escherichia coli* in dark conditions were 15.3 ± 0.6, 14.8 ± 0.5, 14.3 ± 0.7, and 14.7 ± 0.6 mm, respectively. Higher inhibition zones were obtained for all strains as incubated under light conditions at 17.7 ± 0.6, 16.0 ± 1.0, 17.6 ± 0.6, and 16.3 ± 0.5 mm, respectively (Fig. 3A–D). Similarly, the inhibition zones formed against different *Candida* species, *C. albicans, C. glabrata, C. tropicalis,* and *C. parapsilosis* due to the treatment with 400 µg mL^−1^ Se-NPs at dark conditions were 16.3 ± 0.6, 15.7 ± 0.6, 18.0 ± 2.0, and 14.7 ± 0.6 mm, respectively, whereas inhibition zone at 17.7 ± 0.6, 19.7 ± 0.7, 20.3 ± 0.7, and 16.3 ± 0.6 mm, respectively were achieved after incubations under light conditions (Fig. 4A–D). Similar results showed that the highest inhibition zones of 14.0 ± 0.8 mm and 15.0 ± 1.4 mm were recorded for high Se-NPs concentration fabricated by aqueous extract of *Ceropegia bulbosa* against *B. subtilis* and *E. coli,* respectively^25^. Also, the inhibitory effects of Se-NPs fabricated by lactic acid bacteria, *Lactobacillus paracasei* against different species of *Candida* and *Fusarium* were increased by increasing the concentrations^49^. Moreover, Se-NPs synthesized by *Bacillus subtilis* exhibited high antibacterial and antifungal activity in a dose-dependent manner^50^.

To implicate the green synthesized Se-NPs in the pharmaceutical industries, the minimum inhibitory concentration (MIC) values for each tested organism should be determined. The activity of different concentrations (200, 100, 50, 25, 12.5, and 6.25 µg mL^−1^) of Se-NPs was investigated. Data analysis showed that the MIC values for bacterial strain *B. subtilis* was 50 µg mL^−1^ and was 100 µg mL^−1^ for the other bacterial strains under dark conditions. Whereas lower values of MIC were obtained at 12.5, 50, 25, and 50 µg mL^−1^ for bacterial strains of *B. subtilis*, *S. aureus*, *P. aeruginosa,* and *E. coli*, respectively in the presence of light (Fig. 3A–D). Moreover, the MIC values of Se-NPs against *C. albicans, C. glabrata, C. tropicalis,* and *C. parapsilosis* were 50, 50, 25, and 200 µg mL^−1^, respectively in dark conditions and 25, 12.5, 12.5, and 50 µg mL^−1^, respectively in presence of light (Fig. 4A–D). Overall, our study has shown that the fungal species of *Candida* were more sensitive toward green synthesized Se-NPs than bacterial strains. Among prokaryotic strains, the Gram-positive bacteria were more sensitive than Gram-negative bacteria. The obtained data are compatible with those reported for the Phyto-fabricated Se-NPs using fruit aqueous extract of *Emblica officinalis* that exhibited higher antimicrobial activity against fungi than Gram-positive and Gram-negative bacteria^51^.

The NPs size is considered the main factor for their inhibitory effect. The smaller size should easier penetrate the microbial cell wall and cell membrane and hence induces the lysis of microbial cells^52^. Moreover, it can effortlessly interfere with microbial metabolic activities more than larger size NPs. Among these activities, ATP synthesis, respiratory sequence, cell division, and transport chains which ultimately accelerate cell death^51,53^. Therefore, the small Se-NPs size (3–22 nm) formed in the current study is potential responsible for the wide range of antimicrobial activity.

**Figure 5:**
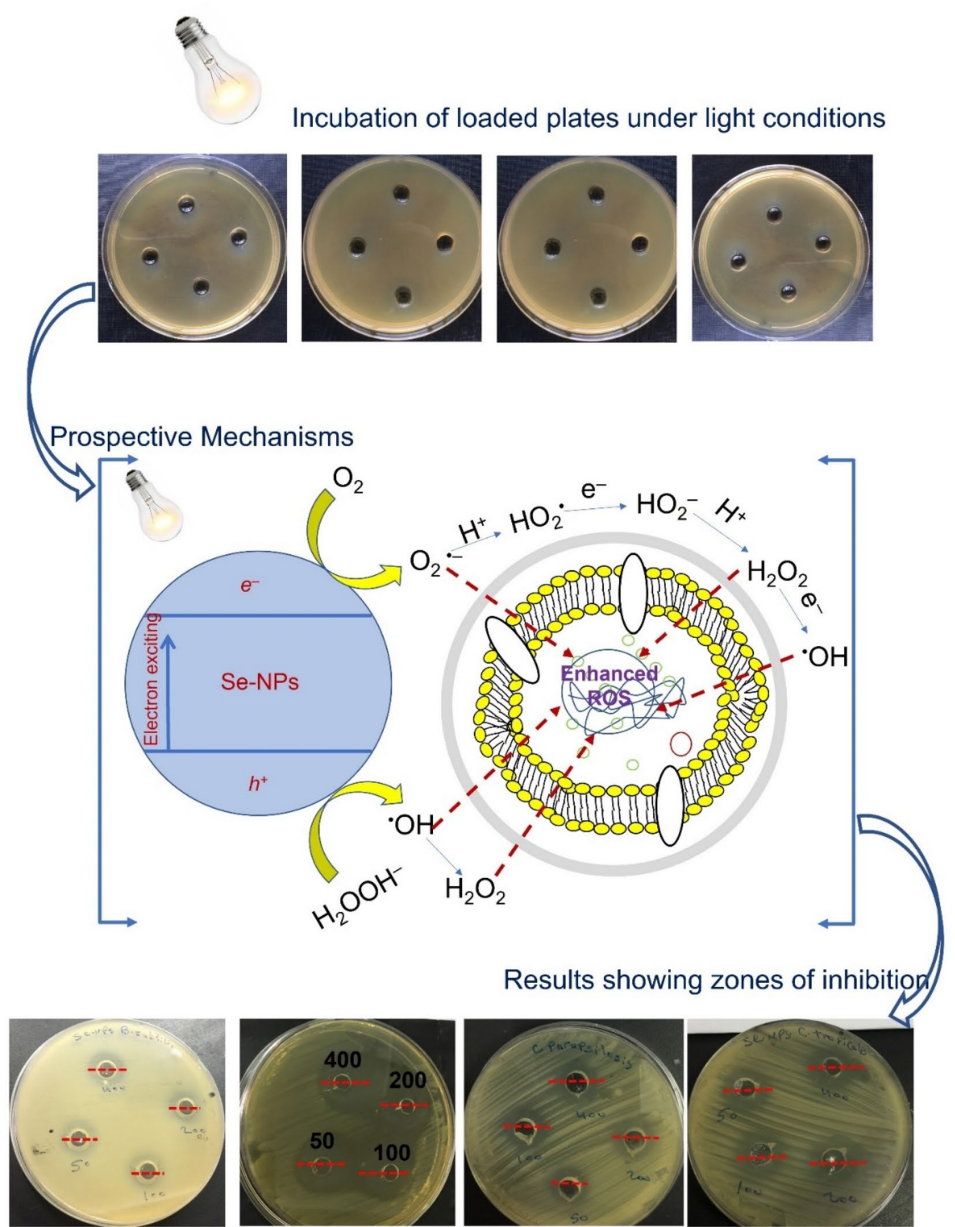
The prospective antimicrobial mechanism of Se-NPs upon exposure to light.

Our study proved that the synthesized Se-NPs have higher antimicrobial activity against Gram-positive than Gram-negative bacterial strains. This may be attributed to the high electrostatic repulsion of synthesized Se-NPs toward negatively charged membrane components and lipopolysaccharide in Gram-negative bacteria. Whereas negative charges are less in Gram-positive bacteria and hence Se-NPs are highly deposited on the surface of Gram-positive bacteria leading to cell death^54,55^. *Candida* especially *C. albicans* are considered the main opportunistic yeast that causes mild to acute diseases in the oral cavity, skin, gastrointestinal, mucous membranes, and urogenital tracts in the low immunity persons, pregnant women, and persons having antibiotic resistance due to misuse for long times^56^. Our study has shown that the synthesized Se-NPs have promising activity against different species of *Candida*. This activity can be related to the efficacy of Se-NPs to inhibit the ergosterol pathways that lead to alteration of sterol profile in the cell wall of *Candida* spp.^57,58^. Also, Se-NPs can be highly accumulated in the *Candida* cell wall by chemisorption, leading to the mixing of the cell wall proteins with selenium and hence destroying the amino acid containing sulfur such as cysteine and methionine^59^. The obtained data showed that the antimicrobial activity of green synthesized Se-NPs was enhanced in the presence of light, and this might be attributed to the production of various toxic free radicals such as O_2_^·−^, ^·^OH, and H_2_O_2_ upon exposure to light that enhance the stresses in the cells as shown in Fig. 5.

### Anticancer activity of Se-NPs

**Figure 6:**
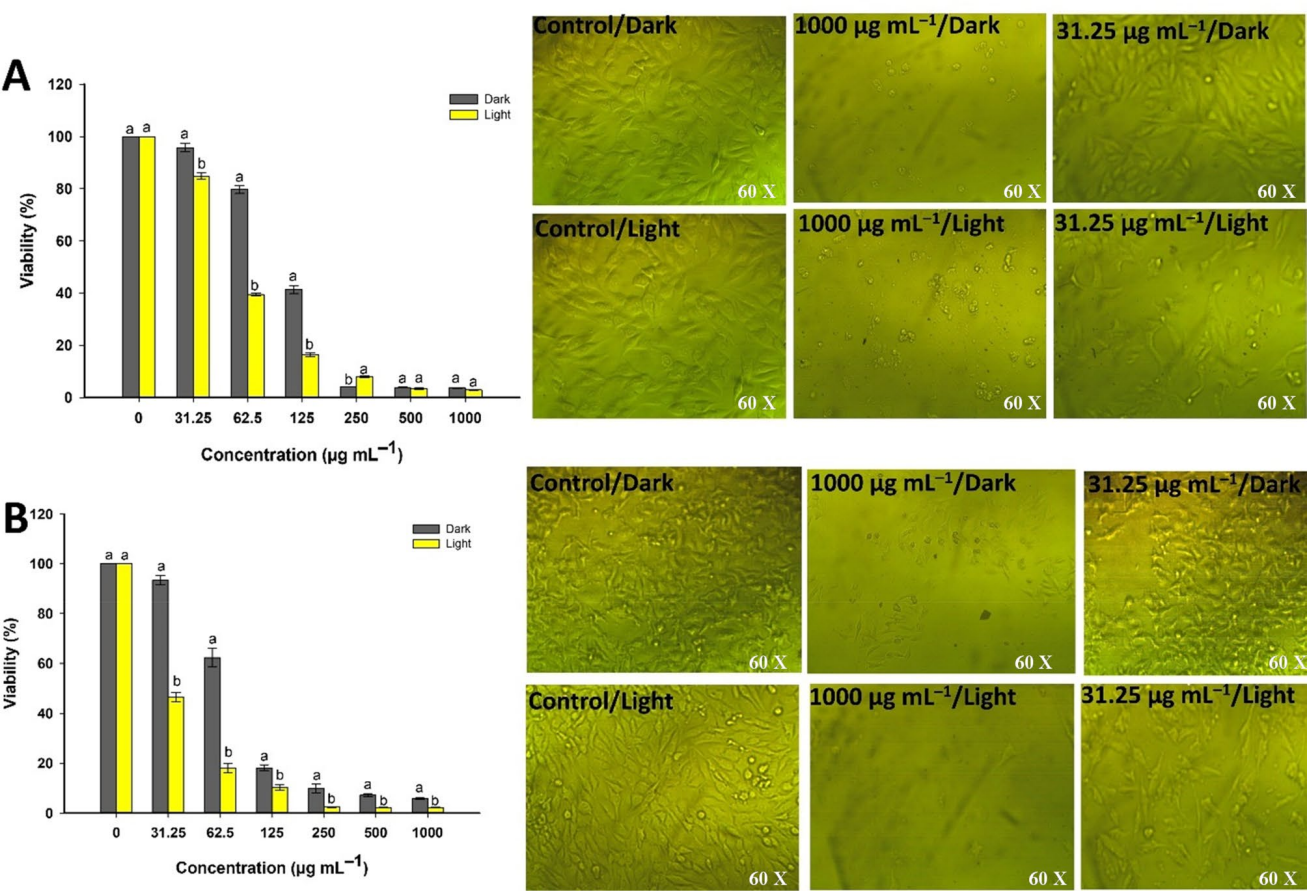
The cell viability and microscopic changes of cancer cells T47D (**A**) and HepG2 (**B**) after treatment with Se-NPs in the presence and absence of light irradiation conditions.

The MTT assay is used as a sensitive method to evaluate the cytotoxic effect of Se-NPs against cancer cell lines in the presence and absence of light irradiations. Data represented in Fig. 6 exhibited that the cell viability of cancer cells was dependent on the NPs concentration and the incubation conditions. Cell viability was decreased by increasing the Se-NPs concentration under light incubation than that under dark condition. Several studies also proved that the effect of NPs against cancer cell lines is concentration-dependent^60–62^.

Under normal incubation conditions (dark conditions), the viability of T47D and HepG2 cells were (3.5 ± 0.1, 3.8 ± 0.3, 4.1 ± 0.02, 41.3 ± 2.7, 79.7 ± 2.6, and 95.8 ± 2.9%) and (5.7 ± 0.6, 7.2 ± 1.1, 9.9 ± 3.1, 18.2 ± 1.9, 62.3 ± 6.4, and 93.4 ± 3.2%) after exposure to 1000, 500, 250, 125, 62.5, and 31.25 µg mL^−1^ of green synthesized Se-NPs, respectively (Fig. 6A,B). These values were decreased upon incubation under light irradiation conditions to be 2.8 ± 0.1, 3.1 ± 0.6, 8.03 ± 0.7, 16.3 ± 1.3, 39.4 ± 0.8, and 84.8 ± 2.9% for T47D cells and 2.2 ± 0.2, 2.3 ± 0.3, 2.4 ± 0.3, 10.4 ± 2.03, 18.1 ± 3.3, and 46.4 ± 3.3% for HepG2 cells, respectively. In similar study, the viability of A549 (lung cancer cells) was decreased to be 70, 45, and 25% after exposure to various concentrations of Se-NPs at 20, 60, and 100 µg mL^−1^, respectively, whereas these values were more decreased in presence of X-ray to be 45, 18, and 5%, respectively^10^.

In the current study, the microscopic examination of the treated cells showed that the monolayer sheet that characterized the epithelial cells were completely or partially lost at high concentration. Other symptoms such as buoyancy, shrinking, a tendency to be spherical or grainy and reduced cell population were also observed (Fig. 6A,B). These symptoms were more noticed in the presence of lights. The cytotoxic effect of NPs can be attributed to their size, smaller sizes are easy to penetrate the cell wall and hence interact with cell components leading to damage of main components such as DNA, proteins, and amino acids^63^. Moreover, light may have a synergistic effect on Se-NPs in enhancing the killing of cancer cells via apoptosis and/or cell arresting mechanisms. Increasing cell killing in presence of light irradiation is attributed to the efficacy of light to generate a high amount of reactive oxygen species (ROS) which harm mitochondria and hence initiate the apoptotic pathway.

Higher activity of Se-NPs was achieved in the presence of light irradiation. Analysis of variance showed that the IC_50_ values were significantly decreased from 109.1 ± 3.8 µg mL^−1^ for T47D cells under dark conditions to 19.7 ± 7.2 µg mL^−1^ under light condition. Whereas the IC_50_ values of Se-NPs against HepG2 cells were 70.4 ± 2.5 µg mL^−1^ and 4.8 ± 4.2 µg mL^−1^ under dark and light irradiation, respectively. The IC_50_ results revealed that the activity of green synthesized Se-NPs as anticancer agents was higher in the HepG2 cell lines compared to T47D. This finding can be attributed to the high expression of purinoceptor in HepG2 cancer cells after treatment of Se-NPs. Compatible with the obtained data, the anticancer activity of ATP-Se-NPs in Caco-2 cell lines was higher than HEK293 cell lines because of high expression of purinoceptor in Caco-2 cells^64^. The obtained data confirmed low values of IC_50_ and the high activity of low doses of Se-NPs in killing cancer cells under light irradiations would be more beneficial from these findings in chemo- and radiotherapeutic for regulating the progression of cancer cells.

### Photocatalytic degradation of methylene blue

**Figure 7:**
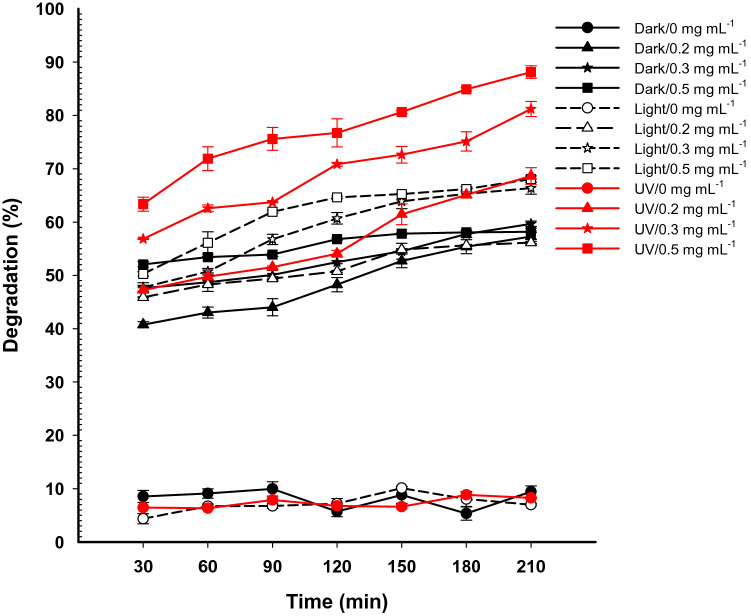
The catalytic activity of different concentrations of Se-NPs (0.2 mg, 0.3 mg, and 0.5 mg/mL MB aqueous solution) for the degradation of methylene blue dye under dark, visible light, and UV-irradiation conditions.

The catalytic activity for degradation of MB using Se-NPs was assessed with different concentrations at different time intervals under dark, light, and UV illumination conditions. Data analysis showed that MB degradation was dependent on Se-NPs dose and contact time. The sorption or catalytic property was achieved on the nano-catalysts surface, the large surface area implies the high sorption activity^65,66^. In the current study, the green synthesized Se-NPs showed a promising catalytic activity due to their small size with large surface area. As shown in Fig. 7, 40.8 ± 0.9%, 47.6 ± 0.8%, and 52.05 ± 0.9% of MB were only degraded under dark conditions after 30 min with 0.2, 0.3, and 0.5 mg mL^−1^ of Se-NPs, respectively as compared with control (8.5 ± 1.9%). The dye degradation was increased gradually by increasing times to reach up to 57.3 ± 2.8%, 59.5 ± 0.9%, and 59.7 ± 0.2% at 0.2, 0.3, and 0.5 mg mL^−1^ Se-NPs, respectively after 210 min (Fig. 7) as compared to the physical degradation without Se-NPs treatment that reached to 9.4 ± 1.9% after 210 min. The obtained data are incompatible with those reported that the fuchsin basic dye did not exhibit any adsorption property after treatment with Se-NPs in dark conditions^62^.

Analysis of variance showed that the degradation efficiency of MB using Se-NPs was increased under light and UV-irradiation conditions. After 30 min of light irradiation, 45.9 ± 0.2%, 47.7 ± 1.5%, and 50.3 ± 0.8% of MB dyes were only decomposed using 0.2, 0.3, and 0.5 mg mL^−1^ Se-NPs, respectively (Fig. 7). The degradation percentages were gradually increased with times to reach 56.1 ± 0.4%, 66.4 ± 1.9%, and 68.1 ± 1.03% after 210 min using 0.2, 0.3, and 0.5 mg mL^−1^ Se-NPs, respectively. Interestingly, the highest degradation of MB dye was accomplished under UV-irradiation conditions (*p* ≤ 0.001). Dye degradation percentages of 65.1 ± 1.1% and 68.6 ± 2.7% were obtained after 180 and 210 min, respectively in the presence of UV-irradiation using 0.2 mg mL^−1^ Se-NPs. These percentages were increased up to 84.9 ± 0.5% and 89.1 ± 2.1%, respectively using 0.5 mg mL^−1^ Se-NPs (Fig. 7). The highest degradation percentages were attained with high amount of nano-catalyst because of increasing the adsorption sites number that exists on the nano-surface or high production of radicals that have a critical role in the degradation of the pollutants^67,68^. The electrostatic attraction of dyes toward NPs is mainly attributed to negatively charged dyes and positively charged NPs^69^.

### Sorption of MB on Se-NPs surface

**Figure 8:**
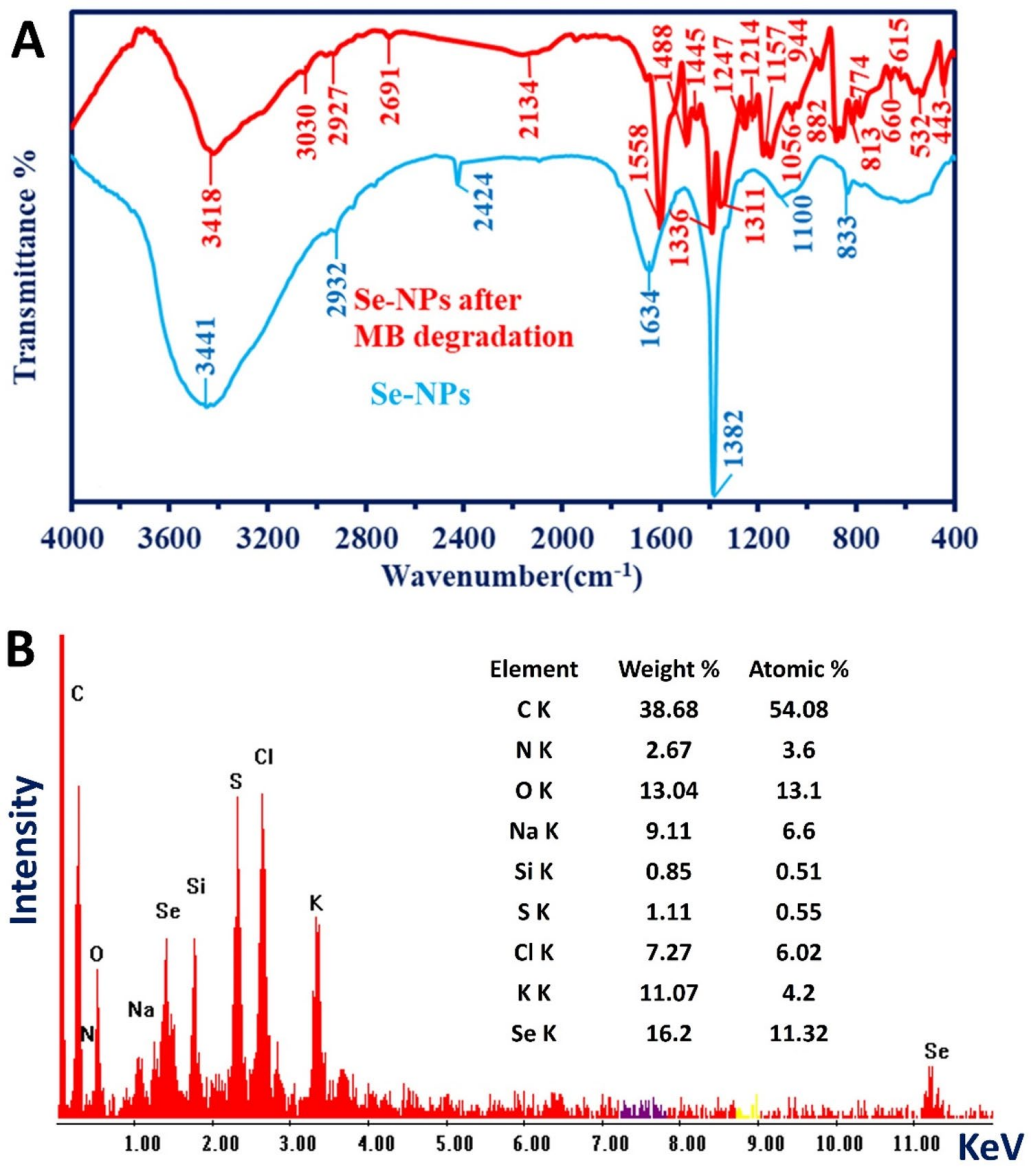
The FT-IR and EDX analysis of Se-NPs after adsorption with MB at optimum conditions (0.5 mg mL^−1^ in presence of UV-irradiation conditions after 210 min).

The FT-IR and EDX were performed for the Se-NPs collected from the optimal MB photocatalytic experiment in presence of UV irradiation after 210 min to confirm the successful sorption of dye over the surface of the catalyst (Fig. 8). Appearance the series of new peaks mainly in the region of figure print, as well as decreasing the intensity of others that were mainly used in sorption was detected as the sorption of MB was performed (Fig. 8A). The OH and N–H stretching bands were decreased with shifts to 3418 cm^−1^ the new peak at 3030 cm^−1^ (associated with N^+^ of the MB moiety) emphasizing the sorption of MB dyes (Fig. 8A)^70^. The peak at 2691 cm^−1^ is related to the S–H stretching band from the metabolite and the broadband at 2134 cm^−1^ for the sulfonic groups^71^. The strong shift of the C=O peak of the Se-NPs composite (appeared at 1634 cm^−1^) to 1558 cm^−1^ is strong evidence for MB sorption. Peaks at 1488 cm^−1^ and 1445 cm^−1^ are assigned to ammonium groups for the MB^72^.

The peak at 1382 cm^−1^ (for Se-NPs) is shifted to 1336 cm^−1^, while other peaks at 1311 cm^−1^ are associated with a peak at 1336 cm^−1^ for the quaternary ammonium group for the MB^72^. Peaks at 1247 cm^−1^, 1214 cm^−1^, and 1157 cm^−1^ are related to C–N of tertiary and quaternary amines (derived from MB) moiety. The peaks at 944 cm^−1^, 882 cm^−1^, and 813 cm^−1^ for C–H of aromatic out-of-plane bend from the MB overlapped with C–O–S bands, while the peak at 774 cm^−1^ for OH out-of-plane bend from polysaccharide moiety. Peaks at 660 cm^−1^ and 615 cm^−1^ for C–S stretching^42,73,74^, and that at 532 cm^−1^ and 443 cm^−1^ for the aryl sulfides^75^. These data provide strong evidence for the adsorption of MB on Se-NPs.

Compatible with FT-IR, the presence of new peaks or change in the weight and atomic percentages of some peaks compared with the original EDX chart of Se-NPs indicates the successful sorption of MB (Fig. 8B). The presence of new peaks such as N with weight and atomic percentages of 2.67 and 3.28% may be originated from MB. Moreover, the increase in weight and atomic percentages of C and Cl peaks from (16.82 and 32.01%) and (5.76% and 3.81%) to (38.68% and 54.08%) and (7.27% and 6.02%), respectively confirmed the successful adsorption of MB over the catalyst. Also, increasing the weight and atomic percentages compared with the original EDX chart indicates the presence of MB on the surface of the catalyst (Fig. 8B).

**Figure 9:**
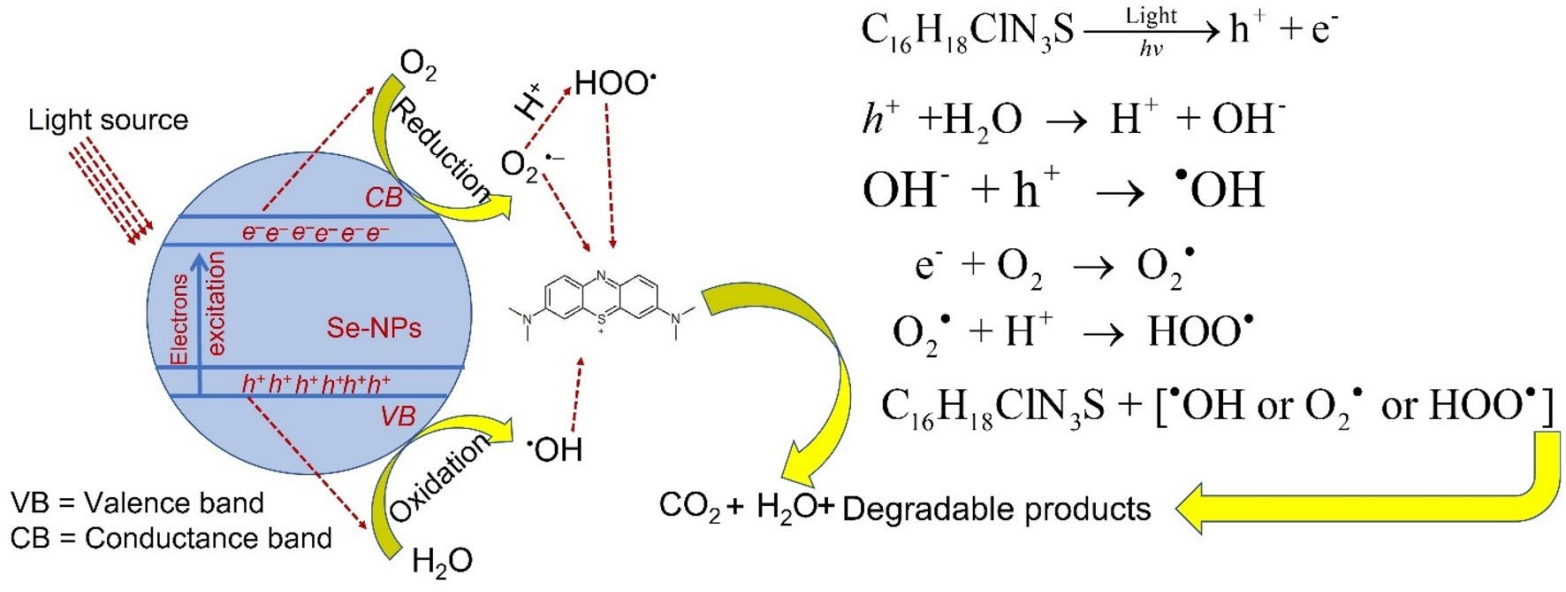
The prospective photocatalytic mechanism for the degradation of methylene blue using green synthesized Se-NPs.

The dye's decolorization and degradation using NPs can be explained by three mechanisms. The first mechanism is the transformation of colored dyes to leuco or white forms (colorless) due to the action of heat (thermochromism), light (photochromism), or pH of the medium (halochromism)^76^. This transformation can be achieved using an oxidation/reduction reaction. The synthesized Se-NPs interact with MB and generate electrons that reduce the central ring of the MB and hence change its conjugative structure to leuco form (colorless) as previously reported^77^. The second mechanism involves the sorption of dyes on the adsorption sites that exist on the NP’s surface^78^. The smaller the size of Se-NPs synthesized in the current study, the larger their surface area, and hence high amounts of dyes can be adsorbed. This mechanism would be mainly used for dye removal under dark conditions. The third mechanism is the decomposition of dyes adsorbed on the surface of NPs due to the electron excitation by light exposure (photocatalyst). Accordingly, the higher activity of synthesized Se-NPs (antimicrobial and photocatalyst) in the presence of light has been achieved. Once the light hits the synthesized Se-NP, the photons of light will be absorbed over the surface of Se-NP that act as a photoactive center for producing the electron–hole pairs. As a results, the electrons are excited and transferred from valance band to conductance band of Se-NPs leaving holes in valance band. After that, the holes (*h*^+^) in valence band interact with H_2_O forming high amounts of hydroxyl (^·^OH) radicals. On the other hand, the excited electrons (*e*^−^) in the conductance band interact with oxygen forming superoxide (^·^O_2_^−^) and hydrogen peroxide (^·^OOH) radicals. In the end, the generated radical species react with MB leading to the degradation of CO_2_, H_2_O, and other degradable products as shown in Fig. 9^23^.

### Reusability of green synthesized Se-NPs

**Figure 10:**
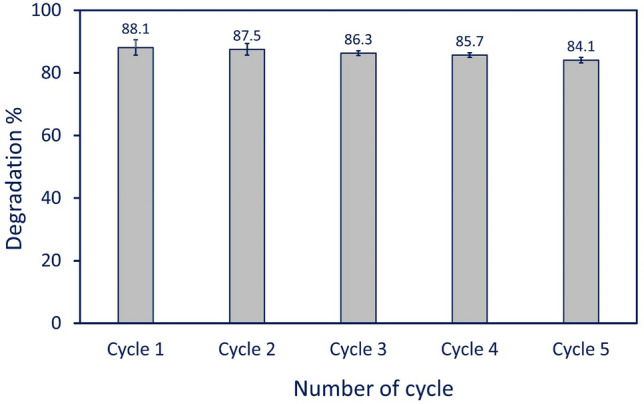
Reusability test of green synthesized Se-NPs for degradation of MB for five consecutive cycles.

The reusability of synthesized NPs in catalytic activity is considered an important factor for industrial applications^79^. Therefore, five sequential cycles were conducted to investigate the efficacy of synthesized Se-NPs. The reusability of Se-NPs was checked under the optimum conditions according to catalytic activity using 0.5 mg mL^−1^ after 210 min under UV-irradiation conditions. At the end of each cycle, the catalytic powder was collected and washed thrice with dH_2_O before being oven-dried at 60 °C for reuse in the subsequent cycle. Data analysis showed the superior stability of Se-NPs in degradation efficiency at different cycles with limited loss in the activity (Fig. 10). Analysis of variance showed that the degradation percentages of MB after the first cycle was 88.1 ± 2.5%. By repeating the photocatalytic activity, almost comparable degradation activities were achieved that decreased by only 4% after five cycles achieving degradation efficiency at 84.1 ± 0.9% (Fig. 10). ZnO-NPs, CuO-NPs, and their composites fabricated by *Penicillium corylophilum* also showed high stability in MB degradation after subsequential ten cycles with loss percentages of 4%^80^.

## Material and methods

### Materials

Sodium selenite (Na_2_SeO_3_) which used as a precursor for selenium nanoparticles synthesis and methylene blue (MB) dye (C_16_H_18_ClN_3_S·xH_2_O) as a model dye for photocatalyst study were analytical grade, purchased from Sigma Aldrich (Darmstadt, Germany). The bacterial strains which used for antibacterial investigation were purchased from American Type Culture Collection (ATCC), whereas *Candida* spp. were clinical strains that isolated and identified by traditional and molecular methods in Microbiology Laboratory, National Research Centre, Dokki, Giza, Egypt. The cancer cell lines designated as T47D and HepG2 were obtained from the Holding Company for Biological Products & Vaccines (VACSERA) Dokki, Giza, Egypt. All reactions were achieved by distilled H_2_O (dH_2_O).

### Endophytic fungal strain

The green synthesis of Se-NPs was achieved using the endophytic fungal strain, *Penicillium crustosum* EP-1. This strain was previously isolated from sterilized leaves of the medicinal plant *Ephedra pachyclada*^37^. The identification of this endophytic fungal strain was achieved by amplification and sequencing of the ITS gene and the obtained sequence was deposited in GenBank under the accession number MN954764.

### Green synthesis of Se-NNPs

Heavy growth one disk (8 mm in diameter) of endophytic fungal strain EP-1 was cultured into sabouraud dextrose broth media (SDB) for 7 days at 30 ± 2 °C. After that, the inoculated SDB was filtrated using Whatman filter paper No. 1 to collect the supernatant (biomass filtrate) which was further subjected to centrifugation at 5000 rpm for 15 min to remove any adhering particles. The collected biomass filtrate was used as a reducing agent for the green synthesis of Se-NPs as follows: 51.9 mg of Na_2_SeO_3_ was mixed well with 10 mL dH_2_O and added to 90 mL of collected biomass filtrate to get a final concentration of 3 mM. The previous mixture was mixed well under stirring conditions, at 38 °C, pH 8, for 1 h., and left overnight in dark conditions. The successful formation of Se-NPs was confirmed by color change to ruby red color. The biomass filtrate without metal precursor was running with the experiment as a control^81^. The resultant was collected and washed thrice with dH_2_O and oven dried at 150 °C for 12 h before using.

### Se-NPs characterization

The as-formed ruby red color of overnight Se-NPs solution was monitored by measuring their absorbance at a wavelength of 200–800 nm to detect the maximum surface plasmon resonance (SPR) of synthesized Se-NPs using a spectrophotometer (JENWAY 6305, Staffordshire, U.K.). The biomass filtrate without metal precursor was used as a blank. The X-ray diffraction (XRD) scan was achieved at two theta scales of 10°–80°. The analysis conditions were achieved at a voltage of 40 kV, a current of 30 mA, and the X-ray radiation source was Cu Ka. The functional groups assigned to different fungal metabolites in biomass filtrate and their role in reducing and capping of Se-NPs were analyzed using Fourier transform infrared (FT-IR) spectroscopy. Approximately, 15 mg of Se-NPs powder was mixed well with KBr to form a disk before being analyzed by FT-IR in a range of 400–4000 cm^−1^ (Agilent system Cary 660 FT-IR model)^8^. Transmission Electron Microscopy (TEM) (JEOL 1010, Japan, 200 kV, X50000) was used to investigate the sizes and shape of green synthesized Se-NPs. A few drops of Se-NPs colloidal solution were loaded on the TEM grid and subjected to vacuum desiccation overnight before being TEM analyzed. The surface morphology and elemental composition of the sample were analyzed by Scanning Electron Microscopy connected with energy-dispersive X-ray [SEM–EDX] (JEOL, JSM-6360LA, Japan). Subsequently, X-ray diffraction (X’Pert PRO, Philips, Eindhoven, Netherlands) was used to investigate the crystalline nature of green synthesized Se-NPs.

### Antimicrobial activity

The antimicrobial activity of green synthesized Se-NPs was investigated against coded bacterial strains represented by Gram-positive bacteria (*Bacillus subtilis* ATCC6633, and *Staphylococcus aureus* ATCC6538), Gram-negative bacteria (*Escherichia coli* ATCC8739, and *Pseudomonas aeruginosa* ATCC9022), and clinical unicellular *Candida* spp. (*C. albicans, C. tropicalis, C. glabrata,* and *C. parapsilosis*) using the agar well diffusion method. The bacterial and unicellular fungi were sub-cultured on Muller Hinton agar (Ready prepared-oxide) and Sabouraud agar media (containing g L^−1^: dextrose, 40; peptone, 10; agar 15; pH was adjusted at 7.0), respectively. After 24 h of incubation, each strain was streaked over a new plate using a sterilized swab, and wells (0.6 in diameter) were made and filled with 100 µL of the prepared Se-NPs concentration. The double-fold concentrations used were 400, 200, 100, 50, 25, 12.5, and 6.25 µg mL^−1^. The loaded plates were kept in the refrigerator for one hour before being incubated at 35 ± 2 °C for 24 h. The results were recorded as a diameter of a clear zone (mm) around each well. The minimum inhibitory concentration (MIC) was determined as the lowest Se-NPs concentration that gives the inhibition zone around the well. Another set of experiments were conducted under the same conditions in presence of light source (Halogen tungsten lamp, 100 W with λ > 420 nm and a light intensity of 2.87 W m^−2^). The distance between light source and plates was 30 cm to investigate the efficacy of light on the Se-NPs activity.

### Anticancer activity

Two cancer cell lines designated as T47D (human breast cancer cell lines) and HepG2 (human liver cell lines) were used to investigate anti-cancer activity of green synthesized Se-NPs by MTT (3-(4,5-dimethylthiazol-2-yl)-2,5-diphenyl tetrazolium bromide) assay method. The selected cancer cell lines were inoculated in 96-well plates (1 × 10^5^ cell/well) and treated with double-fold Se-NPs concentrations (1000, 500, 250, 125, 62.6, and 31.25 µg mL^−1^). A set of treated plates were incubated at 37 °C for 24 h in dark conditions, whereas the second set of treated plates was incubated under the same conditions but in presence of lights (Halogen tungsten lamp, 100 Watt, with λ > 420 nm and a light intensity of 2.87 W m^−2^, the distance between light source and plates was 30 cm). Later, the treated plates were covered with MTT reagent (5 mg mL^−1^ dissolved in phosphate buffer) and incubated at 37 °C for 5 h in presence of 5% CO_2_. Finally, the final product (purple formazan crystals as MTT metabolic products) was dissolved in 10% DMSO. The plates were incubated in dark conditions under agitation for 30 min before measuring the intensity of formed color at 570 nm using ELIZA (Enzyme-Linked Immunosorbent Assay) microplate reader^10^.

The cell viability after treatment with various concentrations of Se-NPs in the presence and absence of a light source was calculated according to the following equation:

1 $${\text{Cell}}\;{\text{viability}}\;\% = \frac{{{\text{Absorbance}}\;{\text{of}}\;{\text{treated}}\;{\text{sample}}}}{{{\text{Absorbance}}\;{\text{of}}\;{\text{control}}}} \times 100$$

### Degradation of dyes

The catalytic efficacy of synthesized Se-NPs was investigated through the degradation of methylene blue (MB) as a model dye. The experiment was achieved in dark, light (100-W, Halogen tungsten lamp), and UV (mercury lamp, λ > 253 nm) irradiation. The concentrations of Se-NPs used were 0.2, 0.3, and 0.5 mg mL^−1^ of MB solution (10 mg L^−1^). Before the experiment, the MB solution containing Se-NPs underwent stirring for 30 min to confirm the absorption/desorption equilibrium of dye on the catalyst surface^23^. After that, the mixture (MB solution and Se-NPs) was incubated in a dark condition under aeration. The previous step was repeated under the same conditions but with exposure to light and/or UV irradiation. At regular interval times, 2 mL of the treatment was withdrawn and centrifugated at 5000 rpm for 10 min. The optical density of clear supernatant was measured using an M-ETCAL spectrophotometer at maximum MB λ_max_ (664 nm). The color removal percentages were calculated using the following equation^82^:

2 $${\text{D}}\;(\% ) = \frac{{{\text{A}} - {\text{B}}}}{{\text{A}}} \times 100$$

where D, is the percentage of color removal; A, is the absorbance at zero-time; and B, is the final absorbance at interval times.

The reusability of Se-NPs as a catalyst for MB degradation was assessed under the optimum condition for five cycles. The catalyst was collected from the first cycle and washed thrice with dis. H_2_O and undergo oven-dry at 50 °C to remove the water content before being reused in the next cycle.

### Statistical analysis

All data in the current study are represented as the means of three independent replicates. Data were subjected to statistical analysis using the statistical package SPSS v17. The mean difference comparison between the treatments was analyzed by *t*-test or the analysis of variance (ANOVA) and subsequently by the Tukey HSD test at *p* < 0.05.

## Conclusion

In this study, Se-NPs were green synthesized using the endophytic fungal strain, *Penicillium crustosum*. The crystallinity nature of as-formed Se-NPs was analyzed using XRD whereas the role of functional groups presents in the fungal extract to fabricate nano-Se was investigated by FT-IR. The shape and size of fungal-mediated Se-NPs were detected by TEM. The qualitative and quantitative composition of the sample was investigated by SEM–EDX. The photocatalytic performance of the biosynthesized Se-NPs exhibited higher activities including antimicrobial, anti-cancer, and the efficient degradation of dyes (methylene blue) than applications under dark conditions. Furthermore, Se-NPs as catalysts showed remarkable stability and reusability and that could be reused at least five times without remarkable loss of activity for degradation of organic dyes. From the obtained results, it can be concluded that the presence of light is one of the influential parameters to promote antimicrobial, anticancer, and catalytic activity of Se-NPs. The importance of this study has gained by the synthesis of Se-NPs through safe method using *Penicillium crustosum* as endophytic strain for the first time to resolve environmental and biomedical issues. Also, the comparative study between the activity of green synthesized Se-NPs in dark and light irradiation conditions will be more interesting for a wide range of researchers and readers.

## Data Availability

The data used to support the findings of this study are available from the corresponding author upon request.

## References

[1] Ying S (2022). Green synthesis of nanoparticles: Current developments and limitations. Environ. Technol. Innov..

[2] Fouda A (2022). Enhanced antimicrobial, cytotoxicity, larvicidal, and repellence activities of brown algae, *Cystoseira crinita*-mediated green synthesis of magnesium oxide nanoparticles. Front. Bioeng. Biotechnol..

[3] Dikshit PK (2021). Green synthesis of metallic nanoparticles: Applications and limitations. Catalysts.

[4] Hamza MF (2021). Synthesis of eco-friendly biopolymer, alginate-chitosan composite to adsorb the heavy metals, Cd(II) and Pb(II) from contaminated effluents. Materials..

[5] Fouda A (2021). An eco-friendly approach to the control of pathogenic microbes and anopheles stephensi malarial vector using magnesium oxide nanoparticles (Mg-NPs) fabricated by *Penicillium chrysogenum*. Int. J. Mol. Sci..

[6] Ahmad A (2020). Zinc oxide-selenium heterojunction composite: Synthesis, characterization and photo-induced antibacterial activity under visible light irradiation. J. Photochem. Photobiol. B.

[7] Zhang D, Ma X-L, Gu Y, Huang H, Zhang G-W (2020). Green synthesis of metallic nanoparticles and their potential applications to treat cancer. Front. Chem..

[8] Amin MA (2021). The potency of fungal-fabricated selenium nanoparticles to improve the growth performance of *Helianthus annuus* L. and control of cutworm *Agrotis ipsilon*. Catalysts.

[9] Dhanjal S, Cameotra SS (2010). Aerobic biogenesis of selenium nanospheres by *Bacillus cereus* isolated from coalmine soil. Microb. Cell Fact..

[10] Cruz LY, Wang D, Liu J (2019). Biosynthesis of selenium nanoparticles, characterization and X-ray induced radiotherapy for the treatment of lung cancer with interstitial lung disease. J. Photochem. Photobiol. B.

[11] Ferro C, Florindo HF, Santos HA (2021). Selenium Nanoparticles for biomedical applications: From development and characterization to therapeutics. Adv. Healthcare Mater..

[12] Abinaya M (2019). Microbial exopolymer-capped selenium nanowires—Towards new antibacterial, antibiofilm and arbovirus vector larvicides?. J. Photochem. Photobiol. B.

[13] Filipović N (2021). Comparative study of the antimicrobial activity of selenium nanoparticles with different surface chemistry and structure. Front. Bioeng. Biotechnol..

[14] Abdo AM (2021). Green synthesis of zinc oxide nanoparticles (ZnO-NPs) by *Pseudomonas aeruginosa* and their activity against pathogenic microbes and common house mosquito, *Culex pipiens*. Materials (Basel, Switzerland)..

[15] Hemeg HA (2017). Nanomaterials for alternative antibacterial therapy. Int. J. Nanomed..

[16] Lin W, Zhang J, Xu J-F, Pi J (2021). The advancing of selenium nanoparticles against infectious diseases. Front. Pharmacol..

[17] Khurana A, Tekula S, Saifi MA, Venkatesh P, Godugu C (2019). Therapeutic applications of selenium nanoparticles. Biomed. Pharmacother. Biomed. Pharmacother..

[18] Ren SX, Zhan B, Lin Y, Ma DS, Yan H (2019). Selenium nanoparticles dispersed in phytochemical exert anti-inflammatory activity by modulating catalase, GPx1, and COX-2 gene expression in a *Rheumatoid arthritis* rat model. Med. Sci. Monitor.

[19] Mariadoss AVA, Saravanakumar K, Sathiyaseelan A, Naveen KV, Wang M-H (2022). Enhancement of anti-bacterial potential of green synthesized selenium nanoparticles by starch encapsulation. Microb. Pathog..

[20] Adeleke JT (2018). Photocatalytic degradation of methylene blue by ZnO/NiFe_2_O_4_ nanoparticles. Appl. Surf. Sci..

[21] Hamza MF (2022). Functionalization of magnetic chitosan microparticles for high-performance removal of chromate from aqueous solutions and tannery effluent. Chem. Eng. J..

[22] Ahmed A (2020). Efficient photocatalytic degradation of toxic Alizarin yellow R dye from industrial wastewater using biosynthesized Fe nanoparticle and study of factors affecting the degradation rate. J. Photochem. Photobiol. B.

[23] Hassanien R, Abed-Elmageed AAI, Husein DZ (2019). Eco-friendly approach to synthesize selenium nanoparticles: Photocatalytic degradation of sunset yellow azo dye and anticancer activity. ChemistrySelect.

[24] Tahir MB, Sohaib M, Sagir M, Rafique M (2020). Role of nanotechnology in photocatalysis. Ref. Module Mater. Sci. Mater. Eng..

[25] Cittrarasu V (2021). Green synthesis of selenium nanoparticles mediated from *Ceropegia bulbosa* Roxb extract and its cytotoxicity, antimicrobial, mosquitocidal and photocatalytic activities. Sci. Rep..

[26] Eid AM (2021). Harnessing bacterial endophytes for promotion of plant growth and biotechnological applications: An overview. Plants..

[27] Alam B (2021). Endophytic fungi: From symbiosis to secondary metabolite communications or vice versa?. Front Plant Sci.

[28] Salem SS (2020). Bactericidal and in-vitro cytotoxic efficacy of silver nanoparticles (Ag-NPs) fabricated by endophytic actinomycetes and their use as coating for the textile fabrics. Nanomaterials.

[29] Vanaja M (2013). Phytosynthesis of silver nanoparticles by *Cissus quadrangularis*: Influence of physicochemical factors. J. Nanostruct. Chem..

[30] Srivastava N, Mukhopadhyay M (2015). Green synthesis and structural characterization of selenium nanoparticles and assessment of their antimicrobial property. Bioprocess Biosyst. Eng..

[31] Joshi SM, De Britto S, Jogaiah S, Ito S-I (2019). Mycogenic selenium nanoparticles as potential new generation broad spectrum antifungal molecules. Biomolecules.

[32] Namvar F (2014). Green synthesis and characterization of gold nanoparticles using the marine macroalgae *Sargassum muticum*. Res. Chem. Intermed..

[33] Awad MA (2022). Mycosynthesis, characterization, and mosquitocidal activity of silver nanoparticles fabricated by *Aspergillus niger* strain. J. Fungi.

[34] Mahendra, R., Alka, Y., Bridge, P. & Aniket, G. Myconanotechnology: a new and emerging science. *Applied mycology*, 258–267 (2009).

[35] Salem SS, Fouda A (2021). Green synthesis of metallic nanoparticles and their prospective biotechnological applications: An overview. Biol. Trace Elem. Res..

[36] Visagie CM (2014). Identification and nomenclature of the genus *Penicillium*. Stud. Mycol..

[37] Khalil AMA (2021). Isolation and Characterization of fungal endophytes isolated from medicinal plant *Ephedra pachyclada* as plant growth-promoting. Biomolecules.

[38] Shin S (2022). Functionalization of selenium nanoparticles using the methanolic extract of *Cirsium setidens* and its antibacterial, antioxidant, and cytotoxicity activities. J. Nanostruct. Chem..

[39] Salem SS (2021). Antibacterial, cytotoxicity and larvicidal activity of green synthesized selenium nanoparticles using *Penicillium corylophilum*. J. Cluster Sci..

[40] Singh N, Saha P, Rajkumar K, Abraham J (2014). Biosynthesis of silver and selenium nanoparticles by *Bacillus* sp. JAPSK2 and evaluation of antimicrobial activity. Der. Pharm. Lett..

[41] Hamza MF (2021). Phosphorylation of guar gum/magnetite/chitosan nanocomposites for uranium (VI) sorption and antibacterial applications. Molecules (Basel, Switzerland)..

[42] Coates J (2006). Encyclopedia of Analytical Chemistry, Applications, Theory and Instrumentation. Infrared Spectroscopy in Analysis of Polymer Crystallinity.

[43] Akter M (2018). A systematic review on silver nanoparticles-induced cytotoxicity: Physicochemical properties and perspectives. J. Adv. Res..

[44] Fouda A, Hassan SE-D, Saied E, Hamza MF (2021). Photocatalytic degradation of real textile and tannery effluent using biosynthesized magnesium oxide nanoparticles (MgO-NPs), heavy metal adsorption, phytotoxicity, and antimicrobial activity. J. Environ. Chem. Eng..

[45] Torres SK (2012). Biosynthesis of selenium nanoparticles by *Pantoea agglomerans* and their antioxidant activity. J. Nanopart. Res..

[46] Sribenjarat, P., Jirakanjanakit, N. & Jirasripongpun, K. Selenium nanoparticles biosynthesized by garlic extract as antimicrobial agent. *Sci. Eng. Health Stud. *22–31 (2020).

[47] Sharma G (2014). Biomolecule-mediated synthesis of selenium nanoparticles using dried *Vitis vinifera* (raisin) extract. Molecules (Basel, Switzerland).

[48] Lashin I (2021). Antimicrobial and in vitro cytotoxic efficacy of biogenic silver nanoparticles (Ag-NPs) fabricated by callus extract of *Solanum incanum* L. Biomolecules.

[49] El-Saadony MT (2021). Selenium nanoparticles from *Lactobacillus paracasei* HM1 capable of antagonizing animal pathogenic fungi as a new source from human breast milk. Saudi J. Biol. Sci..

[50] Abdel-Moneim A-ME (2022). Antioxidant and antimicrobial activities of *Spirulina platensis* extracts and biogenic selenium nanoparticles against selected pathogenic bacteria and fungi. Saudi J. Biol. Sci..

[51] Gunti L, Dass RS, Kalagatur NK (2019). Phytofabrication of selenium nanoparticles from *Emblica officinalis* fruit extract and exploring its biopotential applications: antioxidant, antimicrobial, and biocompatibility. Front. Microbiol..

[52] Zonaro E, Lampis S, Turner RJ, Qazi SJ, Vallini G (2015). Biogenic selenium and tellurium nanoparticles synthesized by environmental microbial isolates efficaciously inhibit bacterial planktonic cultures and biofilms. Front. Microbiol..

[53] Badawy AA, Abdelfattah NAH, Salem SS, Awad MF, Fouda A (2021). Efficacy assessment of biosynthesized copper oxide nanoparticles (CuO-NPs) on stored grain insects and their impacts on morphological and physiological traits of wheat (*Triticum aestivum* L.) plant. Biology.

[54] Huang T, Holden JA, Heath DE, O'Brien-Simpson NM, O'Connor AJ (2019). Engineering highly effective antimicrobial selenium nanoparticles through control of particle size. Nanoscale.

[55] Tran PA (2016). Low cytotoxic trace element selenium nanoparticles and their differential antimicrobial properties against *S. aureus* and *E. coli*. Nanotechnology.

[56] Soliman AM (2021). Green approach to overcome the resistance pattern of *Candida* spp. using biosynthesized silver nanoparticles fabricated by *Penicillium chrysogenum* F9. Biol. Trace Element Res..

[57] Abd-Elaziz AM (2021). Synthesis and characterization of the novel pyrimidine’s derivatives, as a promising tool for antimicrobial agent and in-vitro cytotoxicity. J. Iran. Chem. Soc..

[58] Lv Q-Z, Yan L, Jiang Y-Y (2016). The synthesis, regulation, and functions of sterols in *Candida albicans*: Well-known but still lots to learn. Virulence.

[59] Kieliszek M, Błażejak S, Bzducha-Wróbel A, Kurcz A (2016). Effects of selenium on morphological changes in *Candida utilis* ATCC 9950 yeast cells. Biol. Trace Elem. Res..

[60] Ullah S (2017). Bio-fabrication of catalytic platinum nanoparticles and their in vitro efficacy against lungs cancer cells line (A549). J. Photochem. Photobiol. B.

[61] Khan ZUH (2017). Biomedical applications of green synthesized Nobel metal nanoparticles. J. Photochem. Photobiol. B.

[62] Al Jahdaly BA (2021). Selenium nanoparticles synthesized using an eco-friendly method: dye decolorization from aqueous solutions, cell viability, antioxidant, and antibacterial effectiveness. J. Mater. Res. Technol..

[63] Shiny PJ, Mukherjee A, Chandrasekaran N (2016). DNA damage and mitochondria-mediated apoptosis of A549 lung carcinoma cells induced by biosynthesised silver and platinum nanoparticles. RSC Adv..

[64] Saravanakumar K, Sathiyaseelan A, Zhang X, Park S, Wang M-H (2022). Purinoceptor targeted cytotoxicity of adenosine triphosphate-conjugated biogenic selenium nanoparticles in human colon cancer cells. Pharmaceuticals..

[65] Bhakya S (2015). Catalytic degradation of organic dyes using synthesized silver nanoparticles: A green approach. J. Bioremediat. Biodegred..

[66] Saied E (2021). The catalytic activity of biosynthesized magnesium oxide nanoparticles (MgO-NPs) for inhibiting the growth of pathogenic microbes, tanning effluent treatment, and chromium ion removal. Catalysts.

[67] Li S (2019). Combustion synthesis of porous MgO and its adsorption properties. Int. J. Ind. Chem..

[68] Fouda A, Hassan SE-D, Saied E, Azab MS (2021). An eco-friendly approach to textile and tannery wastewater treatment using maghemite nanoparticles (γ-Fe_2_O_3_-NPs) fabricated by *Penicillium expansum* strain (K-w). J. Environ. Chem. Eng..

[69] Nga NK, Hong PT, Lam TD, Huy TQ (2013). A facile synthesis of nanostructured magnesium oxide particles for enhanced adsorption performance in reactive blue 19 removal. J. Colloid Interface Sci..

[70] Hamza MF (2022). Functionalization of magnetic chitosan microparticles—Comparison of trione and trithione grafting for enhanced silver sorption and application to metal recovery from waste X-ray photographic films. J. Environ. Chem. Eng..

[71] Hamza MF (2022). U(VI) and Th(IV) recovery using silica beads functionalized with urea- or thiourea-based polymers—Application to ore leachate. Sci. Total Environ..

[72] Hamza MF (2020). As(V) sorption from aqueous solutions using quaternized algal/polyethyleneimine composite beads. Sci. Total Environ..

[73] Hamza MF (2022). Functionalized biobased composite for metal decontamination—Insight on uranium and application to water samples collected from wells in mining areas (Sinai, Egypt). Chem. Eng. J..

[74] Hamza MF (2022). Sulfonation of chitosan for enhanced sorption of Li(I) from acidic solutions—Application to metal recovery from waste Li-ion mobile battery. Chem. Eng. J..

[75] Colthup N (2012). Introduction to infrared and Raman spectroscopy.

[76] Witzel, C., Gegenfurtner, K. Memory Color. in *Encyclopedia of Color Science and Technology* (R. Shamey Ed.) 1–12 (Springer Berlin Heidelberg), 10.1007/978-3-642-27851-8_58-9 (2020).

[77] Tripathi R (2020). Biosynthesis of highly stable fluorescent selenium nanoparticles and the evaluation of their photocatalytic degradation of dye. BioNanoScience.

[78] Fouda A (2021). Catalytic degradation of wastewater from the textile and tannery industries by green synthesized hematite (α-Fe_2_O_3_) and magnesium oxide (MgO) nanoparticles. Curr. Res. Biotechnol..

[79] Hamza MF (2022). Grafting of thiazole derivative on chitosan magnetite nanoparticles for cadmium removal-application for groundwater treatment. Polymers.

[80] Fouda A, Salem SS, Wassel AR, Hamza MF, Shaheen TI (2020). Optimization of green biosynthesized visible light active CuO/ZnO nano-photocatalysts for the degradation of organic methylene blue dye. Heliyon.

[81] Abbas H, AbouBaker D (2020). Biological evaluation of selenium nanoparticles biosynthesized by *Fusarium semitectum* as antimicrobial and anticancer agents. Egypt. J. Chem..

[82] Fouda A (2022). *Aspergillus flavus-*mediated green synthesis of silver nanoparticles and evaluation of their antibacterial, anti-candida, acaricides, and photocatalytic activities. Catalysts.

